# Syk kinase is required for collaborative cytokine production induced through Dectin-1 and Toll-like receptors

**DOI:** 10.1002/eji.200737741

**Published:** 2008-02

**Authors:** Kevin M Dennehy, Gerben Ferwerda, Inês Faro-Trindade, Elwira Pyż, Janet A Willment, Philip R Taylor, Ann Kerrigan, S Vicky Tsoni, Siamon Gordon, Friederike Meyer-Wentrup, Gosse J Adema, Bart-Jan Kullberg, Edina Schweighoffer, Victor Tybulewicz, Hector M Mora-Montes, Neil A R Gow, David L Williams, Mihai G Netea, Gordon D Brown

**Affiliations:** 1 Institute of Infectious Disease and Molecular Medicine, Clinical Laboratory Sciences Division of Immunology, University of Cape TownCape Town, South Africa; 2 Department of Internal Medicine and Nijmegen University Center for Infectious Diseases, Radboud University NijmegenNijmegen, The Netherlands; 3 Sir William Dunn School of Pathology, University of OxfordOxford, UK; 4 Tumor Immunology Laboratory, Nijmegen Center of Molecular Life SciencesNijmegen, The Netherlands; 5 National Institute for Medical ResearchLondon, UK; 6 School of Medical Sciences, Institute of Medical Sciences, University of AberdeenAberdeen, UK; 7 Department of Surgery, James H. Quillen College of MedicineJohnson City, USA; 8 Department of Medical Biochemistry and Immunology, School of MedicineHeath Park, Cardiff, CF14 4XN, UK

**Keywords:** C-type lectin, Innate immunity, Macrophage, Syk, TLR

## Abstract

Recognition of microbial components by germ-line encoded pattern recognition receptors (PRR) initiates immune responses to infectious agents. We and others have proposed that pairs or sets of PRR mediate host immunity. One such pair comprises the fungal β-glucan receptor, Dectin-1, which collaborates through an undefined mechanism with Toll-like receptor 2 (TLR2) to induce optimal cytokine responses in macrophages. We show here that Dectin-1 signaling through the spleen tyrosine kinase (Syk) pathway is required for this collaboration, which can also occur with TLR4, 5, 7 and 9. Deficiency of either Syk or the TLR adaptor MyD88 abolished collaborative responses, which include TNF, MIP-1α and MIP-2 production, and which are comparable to the previously described synergy between TLR2 and TLR4. Collaboration of the Syk and TLR/MyD88 pathways results in sustained degradation of the inhibitor of *k*B (I*k*B), enhancing NF*k*B nuclear translocation. These findings establish the first example of Syk- and MyD88-coupled PRR collaboration, further supporting the concept that paired receptors collaborate to control infectious agents.

## Introduction

Originally identified in *Drosophila*, the Toll-like receptors (TLR) consist of a family of at least 11 proteins, which recognize a diverse, but receptor-specific range of microbial structures. Ligand recognition leads to TLR homo- or heterodimerization and the initiation of specific signaling cascades mediated by the intracellular adaptors MyD88 and TRIF 1. This leads to the activation of transcription factors, including NF*k*B, inducing TLR-specific patterns of gene expression. However, the specificity of these responses is incompletely understood and is thought to stem, at least in part, from the association with particular intracellular adaptors, heterodimerization, synergy between selected TLR, and the contribution of other non-TLR pattern recognition receptor (PRR) that are associated with the recognition of specific microbes 1–5. Recently, it has been proposed that complex sets of PRR collaborate to mediate host immune responses to intact microbes 2. For fungi, one such set comprises the β-glucan receptor Dectin-1 and TLR2 5, 6.

Dectin-1 is essential for the innate response to fungal pathogens 7. *In vitro* studies have shown that recognition of fungal β-1,3-glucan by Dectin-1 can induce phagocytosis, phospholipase A2, COX2, the respiratory burst and the production of numerous cytokines and chemokines, including TNF, MIP-2, IL-12, IL-2, IL-10, IL-6 and IL-23 5. Signaling from Dectin-1 is mediated through novel pathways, including an unusual interaction with spleen tyrosine kinase (Syk) which triggers downstream signaling through CARD9 5, 8. While the activation of Syk is sufficient for the induction of the respiratory burst in macrophages and IL-10 and IL-23 in dendritic cells 9–11, the role of this kinase in the induction of the other cytokines and chemokines, if any, is unclear. Furthermore, in macrophages, some of Dectin-1-mediated responses, such as phagocytosis, are Syk-independent, while others, such as the induction of TNF, require collaborative recognition of another undefined fungal component by TLR2, and signaling through the MyD88 pathway 5, 12–14.

How Dectin-1 and TLR2 collaborate to induce proinflammatory cytokines and chemokines is not understood. We show here that this receptor collaboration requires the Syk kinase pathway, utilized by Dectin-1, and that this pathway can collaborate with many other TLR to induce optimal cytokine and chemokine responses. Collaboration of these pathways results in sustained I*k*B degradation and enhanced NF*k*B nuclear translocation. These results demonstrate the importance of receptor collaboration during infection as opposed to receptors functioning in isolation.

## Results and discussion

### Collaboration of Dectin-1 and TLR2 using specific ligands

**Figure 1:**
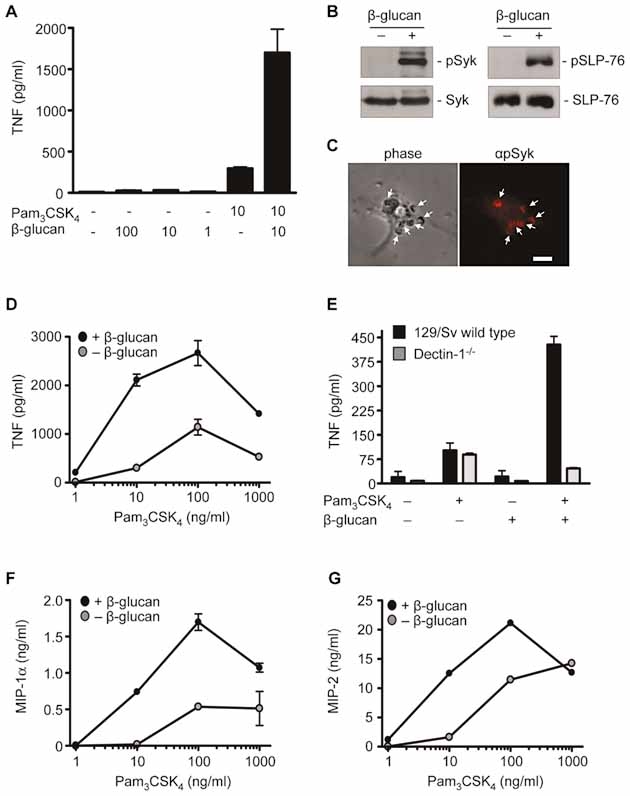
Collaborative induction of pro-inflammatory cytokines through Dectin-1 and TLR2 using specific ligands. (A) Production of TNF by C57BL/6 macrophages stimulated with 100, 10 or 1 μg/mL particulate β-glucan, with or without 10 ng/mL Pam_3_CSK_4_. (B) Western blots showing phosphorylation of Syk, and its substrate SLP-76, upon stimulation of RAW macrophages with 10 μg/mL β-glucan. Data shown are representative of two independent experiments. (C) Activation of Syk in 129Sv macrophages by β-glucan (arrows), as determined by fluorescence microscopy. Left panel, phase image; right panel phospho-Syk image. Scale bar indicates 10 μm. (D) Collaborative production of TNF by C57BL/6 macrophages stimulated with 10 μg/mL particulate β-glucan occurs over a range of Pam_3_CSK_4_ concentrations. (E) Collaborative TNF production by 129/Sv macrophages stimulated with 10 μg/mL particulate β-glucan and 10 ng/mL Pam_3_CSK_4_ is dependent on Dectin-1 expression. Collaborative MIP-1α (F) and MIP-2 (G) production from C57BL/6 macrophages stimulated with 10 μg/mL particulate β-glucan occurs over a range of Pam_3_CSK_4_ concentrations. Data shown are mean ± SD and are representative of at least two independent experiments.

Dectin-1 and TLR2 are known to collaborate to induce cytokine production in response to fungal particles 12, 13. In order to determine the nature of this collaboration, we stimulated thioglycollate-elicited macrophages with highly purified ligands specific for each receptor 15. Stimulation with purified β-glucan failed to induce TNF production, even at high doses (Fig. 1A). Given the surprising lack of response to the purified β-glucan, we measured whether Syk kinase was being activated by this ligand, as an indicator of Dectin-1 activation 9 (Fig. 1B and C). Both Syk, and its substrate SLP-76, were phosphorylated following β-glucan stimulation, indicating that engagement of Dectin-1 and activation of the Syk pathway is not sufficient to induce TNF production in macrophages.

To explore collaborative signaling between Dectin-1 and TLR2, we added sub-stimulatory doses of the TLR2-specific ligand, Pam_3_CSK_4_, alone or in combination with β-glucan (Fig. 1A). While stimulation with the low dose of TLR2 ligand induced low levels TNF, a combination of the two ligands induced high levels of this cytokine, suggesting that the Dectin-1 and TLR2 ligands acted in a synergistic fashion for inflammatory cytokine production. Similar results were also obtained with curdlan, another particulate β-glucan used as a Dectin-1 ligand (data not shown). Synergistic induction of TNF was most evident at lower concentrations of Pam_3_CSK_4_ (Fig. 1D), and was not observed in Dectin-1^–/–^ macrophages (Fig. 1E). Chemokines such as MIP-1α and MIP-2 were also efficiently induced by co-ligation but not by β-glucan alone, indicating that synergistic stimulation of TLR2 and Dectin-1 can induce a variety of macrophage responses (Fig. 1F and G). As TNF and MIP-1α production after TLR2 stimulation never reach the levels obtained after costimulation, regardless of ligand concentration (Fig. 1D and F), these data suggest that such responses are qualitatively different and not simply quantitative shifts in the dose-response curve.

### Dectin-1 and TLR2 collaboration requires MyD88 and Syk kinase-signaling pathways

**Figure 2:**
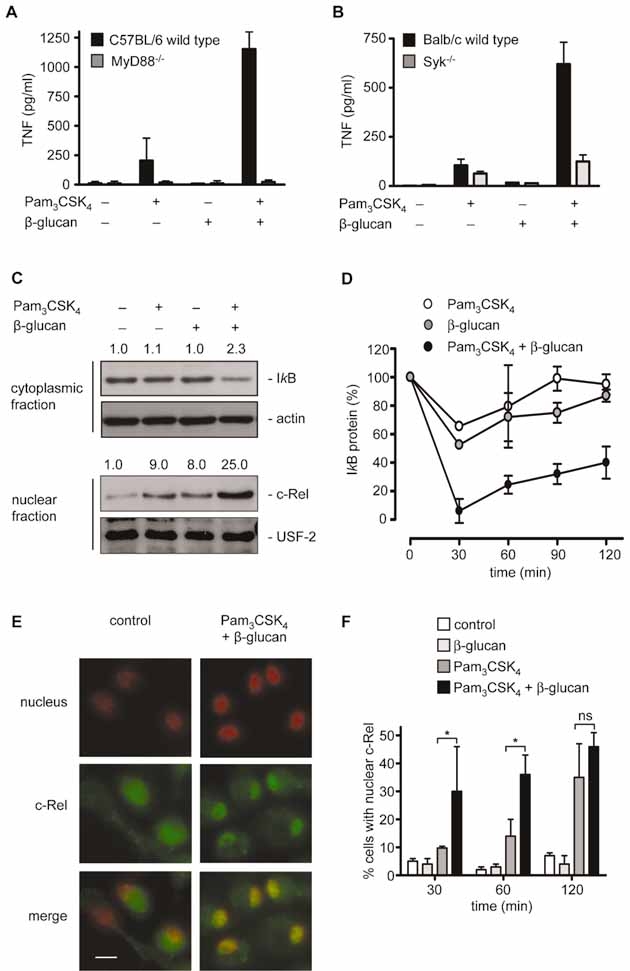
Collaboration of Syk kinase and TLR/MyD88 pathways sustain I*k*B degradation and enhance NF*k*B nuclear translocation. TNF production from C57BL/6 wild-type and MyD88^–/–^ (A) or BALB/C wild-type and Syk^–/–^ (B) macrophages stimulated with 10 μg/mL particulate β-glucan and 10 ng/mL Pam_3_CSK_4_, as indicated. (C) RAW macrophages stimulated with 10 ng/mL Pam_3_CSK_4_ and 10 μg/mL particulate β-glucan. Top: I*k*B degradation after 2 h was assayed by Western blotting. Bottom: localization of NF*k*B c-Rel in nuclear lysates. Numbers above each lane show fold decrease (I*k*B) or increase (cRel) of the relative band intensities of I*k*B and c-Rel, with actin and USF-2 as loading controls, *versus* unstimulated control. (D) I*k*B degradation in RAW macrophages was assayed after the indicated times. Data shown are mean ± SD and are representative of two independent experiments. (E) Nuclear translocation of c-Rel (green) following costimulation of C57BL/6 macrophages with 10 ng/mL Pam_3_CSK_4_ and 10 μg/mL particulate β-glucan for 1 h. Nuclei were stained with Hoechst 33258 (red). Scale bar represents 10 μm. (F) Nuclear translocation of c-Rel was quantified microscopically over time in C57BL/6 macrophages stimulated with 10 ng/mL Pam_3_CSK_4_ followed by 10 μg/mL particulate β-glucan. Data shown are mean ± SD and are representative of two independent experiments.

Collaboration between Dectin-1 and TLR2 in response to fungal particles could occur in two ways. First, Dectin-1 could capture fungal particles and present ligands to TLR2 at the cell surface or in the phagosome, similar to that proposed for CD36 3. Since Syk is not required for binding and phagocytosis of fungal particles by macrophages, such collaboration would be dependant on MyD88 and not Syk, as previously suggested 5, 14. Alternatively, receptor collaboration could require signaling through both receptors and signal integration. In this scenario, collaboration would require both MyD88 and possibly Syk. To test these possibilities, we examined thioglycollate-elicited macrophages derived from wild-type, MyD88^–/–^ and Syk^–/–^ chimeric mice (Fig. 2A and B). In wild-type macrophages from C57BL/6 and BALB/C mice, prominent TNF production was induced by a combination of the two ligands, as before. In contrast, this response was absent in MyD88^–/–^ macrophages (Fig. 2A). However, responses were similarly defective in Syk^–/–^ macrophages, in which the level of TNF production was comparable to that obtained by TLR2 ligation alone (Fig. 2B). These results indicate that efficient induction of specific cytokine responses in macrophages requires integration of the Dectin-1/Syk and TLR/MyD88 signaling pathways, and can thus be defined as synergistic or collaborative responses.

### Receptor collaboration sustains degradation of I*k*B and enhances nuclear translocation of NF*k*B

Both the TLR/MyD88 and Syk pathways couple to I*k*B and NF*k*B activation, ultimately leading to cytokine and chemokine induction 1, 8. However, signaling through the MyD88 pathway alone induces only transient I*k*B degradation, and subsequent transient NF*k*B nuclear translocation in mouse embryonic fibroblasts 16. Similarly, activation of either the TLR2/MyD88 or Dectin-1/Syk kinase pathways alone induced transient I*k*B degradation in a macrophage cell line, with I*k*B protein levels largely recovering after 120 min (Fig. 2C and D). In contrast, costimulation induced sustained I*k*B degradation, which correlated with enhanced nuclear localization of NF*k*B c-Rel (Fig. 2C and D). In order to verify these findings in primary cells, we measured NF*k*B c-Rel nuclear translocation microscopically over time in thioglycollate-elicited peritoneal macrophages (Fig. 2E and F). At early time points following stimulation, the percentages of cells with nuclear c-Rel were significantly greater after costimulation, than after stimulation of either receptor alone (Fig. 2F). However, at later time points there was no significant difference in nuclear c-Rel translocation between the costimulated cells and the cells stimulated with TLR ligand alone. Although we have not defined the point of signal integration, we show that while ligation of either receptor can induce activation of NF*k*B, collaboration of the signals induced by these receptors results in sustained I*k*B degradation and NF*k*B nuclear localization at earlier time points, which is likely to contribute, at least in part, to the enhanced cytokine responses.

### The Syk kinase pathway collaborates with TLR2, 4, 5, 7 or 9

**Figure 3:**
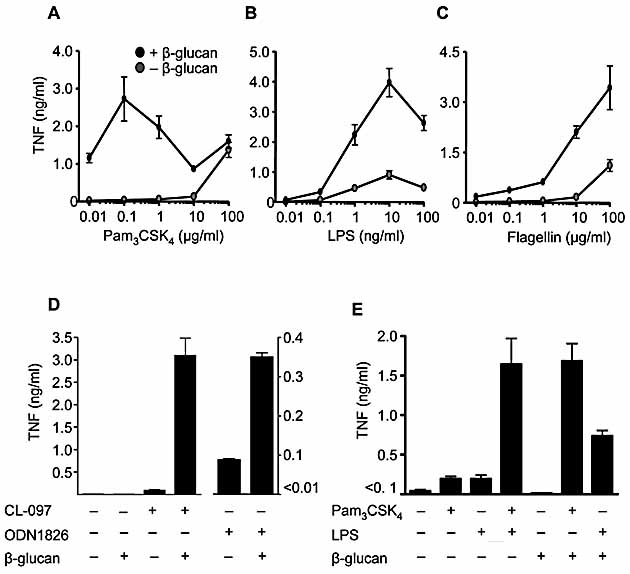
Collaborative responses occur with multiple TLR and are comparable to synergistic responses induced through TLR2 and TLR4. Production of TNF by human peripheral blood mononuclear cells stimulated with or without 10 μg/mL particulate β-glucan and the indicated concentrations of Pam_3_CSK_4_ (A), LPS (B) or flagellin (C). Data shown are mean ± SEM of pooled data from five independent donors. (D) Collaborative TNF responses from C57BL/6 macrophages stimulated with TLR7 (0.2 μg/mL CL-097) or TLR9 (1 μg/mL ODN1826) ligands and particulate β-glucan, as indicated. Data shown are the mean ± SD of one representative experiment of three. (E) Costimulation of C56BL/6 macrophages with 10 μg/mL particulate β-glucan and 10 ng/mL Pam_3_CSK_4_ or 1 ng/mL LPS induces TNF production that is comparable to the synergistic response following TLR2 and TLR4 co-ligation. Shown are the mean ± SEM pooled of data pooled from three independent experiments.

Given that most TLR signal through MyD88, we examined whether the Syk kinase pathway can collaborate with other MyD88-coupled TLR. Stimulation of human peripheral blood mononuclear cells with suboptimal doses of TLR1/2, TLR4 and TLR5 ligands induced low levels of TNF that were greatly enhanced by activation of the Syk pathway with β-glucan (Fig. 3A, B and C). Similarly, coligation of TLR7 or TLR9 with Dectin-1 induced collaborative TNF responses in thioglycollate-elicited macrophages (Fig. 3D). Signaling through TLR/MyD88 is thus a general pathway for collaboration with the Syk kinase pathway.

Receptor synergy has previously been described in innate immunity, notably between TLR that signal through MyD88 and TRIF adaptors respectively 2, 17–19. Collaborative TNF responses after co-ligation of Dectin-1 with either TLR2 or TLR4 in thioglycollate-elicited macrophages were comparable to the previously described synergistic responses between these two TLR (Fig. 3E). As β-glucans have long been known to stimulate anti-infective immunity 5, our results suggest that the activity of these carbohydrates stems from their ability to trigger collaborative responses between Dectin-1 and the various TLR. Similar to specific TLR ligand combinations, the collaborative effects of Dectin-1 and TLR may provide an alternative approach for the development of novel adjuvants.

## Concluding remarks

In this study, we have used defined ligands to explore the nature of the collaborative response induced by the β-glucan receptor, Dectin-1, and TLR2. We show here that the Syk kinase pathway utilized by Dectin-1 is required for this collaboration, and demonstrate that this pathway can also collaborate with TLR4, 5, 7 and 9. The collaborative responses between Dectin-1 and the TLR may be particularly relevant for intact microbes bearing the appropriate combination of ligands. Indeed, the susceptibility to fungal infection of mice deficient in Dectin-1 7, or the downstream signaling pathway 8, despite normal responses to specific TLR ligands, indicates that the defect in these animals is due to the lack of collaborative signaling from the Dectin-1 pathway. Receptor collaboration may also explain the previously observed synergy between mannans, which may be TLR ligands, and β-glucan fungal cell wall components in the response to these organisms 7, 20. These results thus highlight the importance of receptor collaboration during infection as opposed to receptors functioning in isolation, and further support the concept that pairs or sets of receptors collaborate to control infectious agents.

While Syk has been implicated in the modulation of TLR responses in other systems 10, 21–24, the interaction between Dectin-1 and Syk occurs through a novel mechanism 9, which is likely to be representative of other receptors involved in pathogen recognition such as the HIV-1 receptor CLEC-2 and CD66d, a PRR for *Neisseria gonorrhoeae* and *Haemophilus influenzae* 25–28. Recently, it has also been proposed that the adaptor CARD9, which is downstream of Syk in myeloid cells, plays a role in TLR signaling 29. We propose an alternative explanation in which Syk-and CARD9-coupled PRR collaborate with TLR. Stimulation of TLR9 with CpG, for example, induces IL-12p70 production that is dependant on Syk 10, suggesting that an undefined Syk- and CARD9-coupled PRR collaborates with TLR9 in this system. There are therefore likely to be many more examples of Syk-coupled PRR that collaborate with TLR to control infectious agents.

## Materials and methods

### Reagents and mice

All TLR ligands were from InvivoGen (San Diego, CA), and the production of highly purified particulate β-glucan and soluble β-glucan (glucan phosphate) have been described elsewhere 15, 30. BALB/c, C57BL/6 and C57BL/6 MyD88^–/–^, 129/Sv and 129/Sv Dectin-1^–/–^7 mice were obtained from the animal unit of the University of Cape Town. BALB/c Syk^–/–^ chimeric mice were generated by the transfer of Syk^–/–^ fetal liver cells into irradiated BALB/C recipients as described 9. All procedures were approved by the University of Cape Town animal ethics committee.

### Cell stimulation

Murine thioglycollate-elicited macrophages were plated at 10^6^ cells/mL in RPMI medium containing 10% FCS (Gibco) and incubated overnight. Medium was replaced, and cells were stimulated with 10 μg/mL particulate β-glucan or 10 ng/mL Pam_3_CSK_4_, unless otherwise indicated, for 3 h. Human peripheral monocytes were prepared as described 20, 31 and stimulated with 10 μg/mL particulate β-glucan and relevant TLR ligands for 20 h. Cytokine secretion was assayed by ELISA using kits from Becton Dickinson (Mountain View, CA), R&D Systems (Abbingdon, UK) or KOMA Biotechnology (Korea). Data were analyzed using the Student's *t*-test.

### Syk and SLP-76 phosphorylation

RAW 264.7 cells expressing Dectin-1 12 (10^7^cells in 100 μL HBSS) were stimulated with 10 μg/mL soluble β-glucan (glucan phosphate) for 1 min at 37°C before addition of lysis buffer (1% NP40, 25 mM Tris pH 8, 10 mM EDTA, 140 mM NaCl, 5 mM NaF, 1 mM Na_3_VO_4_, 5 mM iodoacetamide) containing protease inhibitors (Roche). Lysate supernatants were incubated for 2 h with 30 μL streptavidin-agarose beads (Fluka) precoated with 25 μM biotinylated Dectin-1 phosphopeptide to precipitate Syk 9 or 30 μL protein G-Sepharose beads (Amersham, UK) precoated with 5 μg SLP-76 mAb. Beads were washed with lysis buffer, boiled in 50 μL SDS-PAGE sample buffer, and samples were Western-blotted and probed with phosphotyrosine mAb 4G10 (Becton Dickinson) and antibodies to Syk or SLP-76 (Santa Cruz Biotechnology, CA). For phospho-Syk staining, 129Sv thioglycollate-elicited macrophages were stimulated with 10 μg/mL particulate β-glucan for 6 min, fixed with paraformaldehyde, blocked and permeabilized with Triton X-100. Cells were stained with anti-phosphoSyk (CellSignalling) followed by donkey anti-rabbit Cy3 (Jackson Immunoresearch) and analyzed by fluorescent microscopy.

### I*k*B degradation and NF*k*B nuclear localization

RAW 264.7 cells expressing Dectin-1 plated at 2 × 10^5^ cells/mL were stimulated with 10 μg/mL particulate β-glucan and 10 ng/mL Pam_3_CSK_4_ for 2 h, or the indicated times, before lysis and Western blotting of whole-cell lysates with antibodies to I*k*B or actin (CellSignaling, MA) as a loading control. Nuclear lysates were prepared after 20 h stimulation as described 32, followed by Western blotting with antibodies to NF*k*B c-Rel or USF-2 (Santa Cruz Biotechnology) as a loading control. Band intensities were quantified using NIHImage software.

### NF*k*B nuclear translocation

C57BL/6 thioglycollate-elicited macrophages plated at 2 × 10^5^ cells/mL were stimulated with 10 ng/mL Pam_3_CSK_4_ for 90 min followed by 10 μg/mL particulate β-glucan for 60 min, fixed with paraformaldehyde, blocked and permeabilized with Triton X-100. Cells were stained with Hoechst and NF*k*B c-Rel antibody, followed by anti-rabbit IgG-Cy6 (Jackson laboratories, USA) and analyzed by fluorescent microscopy. Three independent fields containing >100 cells were counted for each stimulation type, and data were analyzed using the Student's *t*-test.

## Abbreviations:

PRR: pattern recognition receptor
Syk: spleen tyrosine kinase
